# Investigation into the safety, and serological responses elicited by delivery of live intranasal vaccines for bovine herpes virus type 1, bovine respiratory syncytial virus, and parainfluenza type 3 in pre-weaned calves

**DOI:** 10.3389/fvets.2024.1283013

**Published:** 2024-02-23

**Authors:** Anna Flynn, Catherine McAloon, Katie Sugrue, Ricki Fitzgerald, Cara Sheridan, Bosco Cowley, Conor McAloon, Emer Kennedy

**Affiliations:** ^1^Teagasc, Animal & Grassland Research and Innovation Centre, Fermoy, Ireland; ^2^School of Veterinary Medicine, University College Dublin, Dublin, Ireland; ^3^MSD Animal Health, Dublin, Ireland

**Keywords:** BRD, pneumonia, bovine, vaccination, maternally derived antibodies

## Abstract

Despite the fact that pneumonia remains a leading cause of mortality and morbidity in pre-weaned calves, relatively little is known regarding the effects of the concurrent administration of intranasal pneumonia virus vaccines, particularly in calves with high levels of maternally derived antibodies. The objective of this study was to use a cohort of 40 dairy and dairy-beef female and male calves (27 females and 13 males) to determine serological responses to concurrent administration at 3 weeks of age (22 ± 4.85 days) of two commercially available intranasal (IN) vaccines for the viruses: bovine respiratory syncytial virus (BRSV), bovine herpes virus 1 (BoHV-1), and parainfluenza-3-virus (PI3-V). The study groups were as follows: (i) Bovilis IBR Marker Live only® (IO), (ii) Bovilis INtranasal RSP Live® only (RPO), (iii) Concurrent vaccination with Bovilis IBR Marker Live® & Bovilis Intranasal RSP Live® (CV), and (iv) a control group of non-vaccinated calves (CONT). The calves’ serological response post-IN vaccination, clinical health scores, rectal temperatures, and weights were measured. Data were analyzed in SAS using mixed models and logistic regression. The CV calves had an average daily weight gain (ADG) of 0.74 (±0.02) kg, which was similar to CONT (0.77 ± 0.02 kg). Despite no significant differences in the antibody levels between study groups 3 weeks post-IN vaccination, following the administration of subsequent parenteral injections in the form of Bovilis Bovipast RSP®(antigens; inactivated BRSV, inactivated PI3-V, inactivated *Mannheimia haemolytica*) and Bovilis IBR Marker Live®, the antibody levels of the BRSV and PI3-V increased in both the CV and RPO study groups. Concurrent vaccination resulted in no increase in fever and no difference in health scores when compared to CONT.

## Introduction

Bovine respiratory disease (BRD) is one of the primary causes of morbidity and mortality in cattle worldwide (1, 2). Pneumonia is the general term used to describe respiratory infections that cause inflammation of the lung tissue and airways (3). In Ireland, pneumonia remains the leading cause of death in calves between 1 and 12 months of age and the second most prevalent cause of death in animals less than a month of age (4). While the true extent of subclinical pneumonia cases on farms is still largely unknown, some studies have found that the prevalence of cases can be as high as 67% (5). Bovine respiratory disease often develops as the result of a synergistic infection, comprising both bacterial and viral pathogens. Among the viruses most commonly associated with pneumonia are bovine respiratory syncytial virus (BRSV), parainfluenza type 3 (PI3-V), bovine coronavirus, and bovine herpes virus type 1 (BoHV-1), which cause infectious bovine rhinotracheitis (IBR) (6, 7). In calves, even non-fatal BRD episodes have lifelong ramifications on lung function and animal productivity (8–10).

For dairy heifers, experiencing a clinical episode of pneumonia in calfhood has been associated with an increase in age at first calving (11), as well as a reduced likelihood of completing their first lactation (12); even if heifers do complete this first lactation, their milk yields can be reduced (11–13). In beef cattle, growth performance is impaired by BRD, final carcass weights are lower, and days to finishing are increased following recovery from BRD (14–16). With such a high prevalence, it is understandable that treatment for pneumonia is responsible for a large proportion of the antimicrobial usage in calf rearing (17–20). This often metaphylactic use of antibiotics is a topic of increasing public concern as it is known that metaphylactic antibiotic use contributes to the emergence of antibiotic-resistant bacteria (21, 22). Vaccination in combination with improved animal husbandry and management practices is a key tool that should be employed to reduce dependency on antimicrobials by limiting the spread of BRD (23–26).

Studies have found that beef calves are particularly susceptible to BRD during stressful events such as the post-weaning period or following transport (27). While in dairy heifers, management practices such as milk feeding regime and calf housing have been identified as potential influences for the development of BRD (1). In both systems, delivering vaccine induced immunity to calves prior to weaning, presents a challenge. Vaccination of the neonatal calf, though often recommended, can be limited in its efficacy (28, 29), which is because, in the face of high levels of maternally derived antibodies (MDAs), an antigen is compromised in its ability to induce development of specific immunity to the virus in question due to the protective masking effect of the MDAs, cytokines, and cells transferred from the dam to the calf in the colostrum (23, 28, 29). At birth, calves are agammaglobulinemic and so are entirely dependent on this passive immunity to activate and regulate their immune responses to fight infections (30). As such, it is crucial that calves are fed colostrum of a high enough immunoglobulin concentration to ensure adequate passive transfer and to protect them from infections such as BRD. It is also known that there is an increased risk for the development of infectious diseases including BRD in calves between 2 and 4 weeks of age as maternally derived passive immunity wanes, and a number of reviews have suggested that intranasal (IN) vaccination offers a potential strategy to negate the MDA masking effect (28, 31, 32).

In herds with a history of pneumonia, it is often recommended that calves are vaccinated for BRSV/PI3-V and or IBR (BoHV-1) in their first year of life, and these vaccines may be live-attenuated or inactivated (killed) viruses. It is postulated that intranasally delivered live vaccines are more suitable than killed parental vaccines for administration to neonatal calves because live IN vaccines are believed to induce a localized mucosal immunity, even in the face of maternally derived antibodies. It is speculated that it is common practice on farms to administer multiple live IN pneumonia vaccines together, likely for the ease of animal handling. However, this practice is currently unlicensed as there is a paucity of knowledge on the effects of delivering these intranasal pneumonia vaccinations concurrently. Therefore, it is appropriate that the effects of IN vaccination of neonates should be further investigated (10). Studies on the serological response to IN vaccinations for pneumonia in young calves with high levels of MDAs are also somewhat limited, which may be because historically most initial vaccine efficacy evaluations were carried out on colostrum-deprived or seronegative calves (33, 34). The findings of those studies that have been undertaken on IN vaccination in seropositive calves to date have shown how it may not result in seroconversion but instead may induce a protective localized effect at the mucosal sites of the respiratory tract (35–37).

The objective of our study was to determine the serological response elicited in the face of MDAs following concurrent administration, at 3 weeks of age, of two commercially available IN vaccines, Bovilis® IBR Marker Live and Bovilis® INtranasal RSP® Live, for the viruses BoHV-1 and BRSV and PI3-V, respectively. We also aimed to investigate whether concurrent vaccination had any effect on calf average daily weight gains. The first hypothesis of this study is that concurrent vaccination will elicit a detectable serological response similar to individual vaccine administration at 21 days post-IN delivery. Finally, we also hypothesize that concurrent vaccination will not affect calf growth rates.

## Materials and methods

### Ethics statement

Ethics approval to complete the study was granted by the Teagasc Animal Ethics Committee (TAEC2020-278), and a license was granted by the Health Products Regulatory Authority (Clinical Field Trial License Number: CT10452/002).

### Study groups and measurements

This study was carried out on the Teagasc Dairygold Farm, Kilworth, Co. Cork, Ireland. Eighty-six calves born between the 7th and the 22nd of February 2021 were blood-sampled to obtain the antibody levels for BoHV-1, BRSV, and PI3-V. Colostrum fed calves were included in the study to test vaccine responses in the face of maternally derived antibodies. From this cohort, the 40 calves with the lowest levels of maternally derived antibodies for the viruses BoHV-1 (Range = 144.88–156.60 ± 2.69 SP), BRSV (Range = 35.83–108.72 ± 16.02% Pos), and PI3-V (Range = 23.50–99.75 ± 13.69 c S/P) were selected for inclusion in the study groups. As the trial took place on a dairy research farm herd, the farm’s pre-calving vaccination protocols, colostrum management, and biosecurity were extremely stringent, potentially resulting in higher passive transfer of maternal antibodies to calves than what would be achieved on commercial farms. Therefore, to better represent the immune status of calves typical of the Irish national herd, 40 calves with the lowest levels of maternally derived antibodies on farm were chosen. Sample size calculations were conducted based on the expected difference in serological results between the experimental groups. These expected differences were identified for BRSV (±47.9% Pos), PI3-V (±47 c S/P), and BoHV-1 (±30.5 SP) based on data generated by Barry et al., with values from calves aged 1 to 3 months (38). Ten calves per study group were deemed to provide sufficient power for all virus antibody testing. These calves (*N* = 40, 27 females and 13 males) were blocked based on birthweight (33.5 ± 7.09 kg), date of birth (14th Feb 2021 ± 4.85 days), and levels of maternally derived antibodies specific to BoHV-1, BRSV, and PI3-V. The animals were then randomly allocated across four study groups, with each group containing 10 calves. The calves were bred from both dairy and beef sires, with the total cohort consisting of 30 dairy calves [Holstein-Friesian (HF), Jersey (JE) and Jersey Holstein-Friesian Cross (HF x Jersey) and 10 dairy-beef calves (HF x Belgian-Blue) (BBX), HF x Aberdeen-Angus (AAX), HF x Charolaise (CHX), HF x Limousine (LMX)].

The four study groups were as follows:

1. Vaccinated with Bovilis IBR Marker Live® (IO).
2. Vaccinated with Bovilis INtranasal RSP Live® (RPO).
3. Vaccinated concurrently with both Bovilis INtranasal RSP Live® and Bovilis IBR Marker Live® (CV).
4. Control not vaccinated—administered with diluent for Bovilis INtranasal RSP Live® and Bovilis IBR Marker® Live (CONT).

During the observational period of the study, those responsible for the daily husbandry of the calves were blinded to the vaccines administered to each group, and this was known only by an impartial body and the veterinary surgeon who administered the doses. No other vaccines were administered to the groups within a 14-day period before or after each vaccination so as to prevent any misattribution of possible side effects. Calves that required individual veterinary treatment with antibiotics or anti-inflammatory drugs were removed from the trial.

### Vaccination

All the calves were vaccinated by a veterinarian during a single visit at an average age of 22 ± 4.9 days. The IN vaccines consisted of the lyophilized modified live-attenuated virus(es) (MLV) with a diluent. The active substances contained in the Bovilis INtranasal RSP Live® and Bovilis IBR Marker Live® vaccines were live bovine respiratory syncytial virus (BRSV), strain Jencine-2013: 5.0–7.0 log10 at a 50% tissue culture infective dose (TCID50), live bovine parainfluenza virus type 3 (PIV-3), strain INT2-2013: 4.8–7.0 log_10_ TCID50, and live bovine herpesvirus type 1 (BHV-1), strain GK/D (gE¯): 10^5.7^–10^7.0^ TCID50, respectively. These vaccines were stored at 2–8°C before use. Immediately prior to administration, the vaccines were reconstituted by transfer of the solvent to the vial with the lyophilizate using a syringe and needle and then re-suspended by shaking. Automatic vaccination guns (MSD Animal Health, Ireland) were used to administer a single dose of 2 mL (1 mL in each nostril) to the calves. The device was calibrated using a diluent, and its nozzles were changed between each animal. The CV group received both vaccines in this manner, separately and immediately after one another. The CONT were given the diluents for both Bovilis INtranasal RSP Live® and Bovilis IBR Marker Live® administered in the same manner as outlined above as a placebo.

Eighty days after the initial IN vaccination, all calves enrolled in the study received parenteral vaccinations for all three viruses, which consisted of a 2-ml intramuscular dose of Bovilis IBR Marker® Live and additionally a 5-ml dose of Bovilis Bovipast RSP®, subcutaneously. This 5-ml dose of Bovilis Bovipast RSP® contained inactivated bovine respiratory syncytial virus, strain EV908 10^4.7^–10^5.45^ at TCID50, inactivated Parainfluenza-3-Virus, strain SF-4 Reisinger 10^3.54^–10^4.85^ TCID50, inactivated *Mannheimia haemolytica* A1, strain M4/1 10^4.24^–10^5.0^ cells, and the adjuvants aluminum hydroxide B (37.5 mg), quil A (Saponin) 0.189–0.791 mg, and the excipient thiomersal: 0.032–0.058 mg. These parental vaccines were administered so as to assess the response of the different study groups to a subsequent booster vaccination.

### Blood sampling and ELISA antibody analysis

Calves were blood-sampled via a jugular vein; 10 mL samples were drawn using a 20-G needle into a vacutainer tube that contained no coagulant or preservatives (both BD, Vaud, Switzerland). The calves were blood-sampled 5 days before IN vaccination (Day −5), then again on the day of IN vaccination (Day 0), for the third time 3 weeks after IN vaccination (Day 21), and for the final time 2 weeks after the parenteral vaccine administration (Day 94). Following collection, all blood samples were refrigerated between 1°C and 5°C and immediately transported to a commercial laboratory for analysis (Enfer Scientific, ULC Newhall, Naas Co. Kildare). The blood serum was used to determine the levels of specific antibodies for each of the viruses. Values for specific BoHV-1, PI3-V, and BRSV antibodies were determined using commercially available indirect ELISA test kits, and serum was diluted according to kit manufacturer’s instructions. The test kits used were IDEXX IBR Individual Ab Test, IDEXX PI-3 Ab Test (both IDEXX Europe B.V, The Netherlands) and SVANOVIR® BRSV-Ab (SVANOVIR INDICAL AB, Sweden). The optical density of the samples was read at 450 nm with a Tecan Sunrise® Absorbance Reader, and Magellan software was then used to define positive and negative control values according to a standard curve (Tecan Trading AG, Switzerland). The corrected optical density absorbance-based semi-quantitative units were expressed as S/P % (sample to positive ratio) for IBR, %P (percent positive) for BRSV, and c S/P % (corrected sample to positive ratio) for PI3-V.

These ELISA optical density (OD) units were calculated from OD readings according to the following formulas from the test kit instruction manuals.

- BRSV; $\left(\%P=\left(\frac{OD_{Corr\left(SampleorNegativeControl\right)}}{OD_{Corr\left(PositiveControl\right)}}\right)\;x\;100.\right)$
- where BRSV % *p* ≥ 10 = Positive.
- IBR; $\left(S/P\%=\left(\frac{\left(\begin{array}{l} OD\;Sample\\ -OD\;NegativeControl\;\bar{x}\; \end{array}\right)}{\left(\begin{array}{l} OD\;PositiveControl\;\bar{x}\\ -OD\;NegativeControl\;\bar{x} \end{array}\right)\;}\right)\;x\;100.\right),$
- where % S/ *p* > 50 = Positive.
- PI3-V; $\left(c\;S/P\%=(0.8\;(100\;x\;\left(\frac{NE}{NE{\bar{x}}_{PostiveControl}}\right)\right)\;,$
  ((NE=(ODcontrolwell−ODcoatedwell)).
- where c S/P % ≥ 20 = Positive.

### Health scoring

Calves were health scored for 9 continuous days, for 3 days in advance of IN vaccination and for 6 days following IN vaccination. Calves were health scored again for 3 days following parenteral vaccination on Day 80. These health scores were taken according to a modified calf health scoring system devised by Barry et al. (38, 39). This scoring involved a comprehensive evaluation of each animal’s health status, including detailed observations of respiratory rate, coughing, fecal cleanliness, navel and urogenital tract characteristics, nasal discharge, and ocular discharge, ear position, mobility, dehydration, and demeanor, interest in surroundings, and rectal temperature. Calves were scored from zero to three on each of these aspects of health, with zero representing normal and three representing the most severely affected. Calves were scored at the same time each day, an hour after morning feeding. Following cessation of health scoring, animals were monitored daily between feedings for any changes in demeanor or abnormalities, including evidence of poor form, ill thrift, diarrhea, nasal or ocular discharge, or reduction in milk intake.

In addition to health scoring, all animals were observed daily for signs of poor health, and episodes of illness were recorded and treated as necessary by the farm manager/veterinarian. Throughout the study, bouts of nutritional and infectious diarrhea were treated by oral rehydration therapy.

Three calves were withdrawn from the study following veterinary intervention for gastrointestinal illnesses, unrelated to vaccine grouping.

### Calf weights

All calves were weighed weekly for the duration of the study (TruTest XR 3000, Tru-test Limited, Auckland, New Zealand).

### Animal husbandry

All calves were removed from their dam within 1 h of birth. The calves were then weighed, tagged, and moved to individual pens. Before feeding, all of the collected colostrum were tested using a Brix Refractometer (HI 96801, Hanna Instruments, Woonsocket, RI). Only colostrum with a value of >22% Brix, equivalent to >50 mg/mL IgG (40), was fed to calves. Calves were fed 3 L of colostrum within an hour of birth from a single dam, not necessarily their own (41). This was given as one feed via bottle and teat, with a stomach tube used only if a calf refused to drink the colostrum voluntarily.

The calves remained in individual pens for 2 days and received four further feeds of transition milk. Following this, calves were moved into a general group pen of approximately 20 calves in the main calf house. Once randomized, the selected 40 experimental calves were moved into their group pens. These pens were designed to ensure calves had no shared airspace or nose to nose contact with animals from the other study groups. Solid concrete dividing partitions and alternating vacant pens were in place to ensure that each of the study groups were kept a minimum of 5 m apart so as to prevent the possible spread of the live vaccine strains to the alternative study groups. Pens were straw bedded to a depth of 15 cm, and straw was topped up daily and fully cleaned out every 3 days. Each calf had a minimum space allowance of 1.7m^2^ with an additional outdoor feeding area. Ventilation was passive in the calf houses. Tiny Tag data loggers were used to record atmospheric temperature and relative humidity (Gemini Data Loggers Ltd., UK).

All calves were fed 3 L twice daily (6 L/day) with 26% crude protein milk replacer (Volac Heiferlac Instant, Volac, Hertfordshire, United Kingdom), which was mixed at a reconstitution rate of 15% through 10 teat compartmentalized feeders (JFC Manufacturing, Tuam Co. Galway, Ireland). To prevent the spread of the live vaccine virus strains from saliva between study groups, each group had a separate 10 teat compartmentalized milk feeder. The calves were gradually weaned, simultaneously, over a 10-day period and were fully weaned at an average age of 11.7 (±0.68) weeks.

Calves had access to *ad libitum* water and concentrates (18% crude protein, ingredients; barley, soya meal, sugar beet pulp, distiller’s grains, rape seed meal, and maize; Sweet Start Calf Starter Pencils, Southern Milling, Cork, Ireland). At weaning, all calves were consuming >1 kg of concentrates per day. Following weaning, the concentrate calves offered were changed (17% crude protein, ingredients; barley, distillers dried grain, maize, sugar beet pulp, rape seed meal, oats, soy hulls, palm kernel, soya (bean) meal Super Calf Rearer Nuts, Southern Milling, Cork, Ireland).

Biosecurity protocols included disinfecting boots and overclothing and changing gloves when moving between pens. Three weeks post-IN vaccination, the calves were removed from their respective group isolation pen and housed together. After 2 weeks, the calves were moved outdoors to pasture as a single group and remained separated from other animals on the farm until the end of the study.

### Statistical analysis

All data analyses were performed using SAS (2013) version 9.4 (SAS Institute Inc., Cary, NC, USA). Calf was considered as the experimental unit. Normality was assessed using PROC UNIVARIATE. Dependent variables were found to follow a normal distribution pattern. Due to a wide range in birthweights in the data, birth weights were centered by breed class (dairy or dairy-beef). A mixed model (PROC MIXED) was used to determine whether group had an effect on weight data and ELISA OD unit values for IBR, BRSV, and PI3-V. Least square means and interactions were examined between significant variables in each mixed model. Study group, breed class, sex, and time from IN vaccination were included as categorical variables. The final models for weight data and ELISA OD unit values comprised of the interaction between study group and time since vaccination in addition to breed class (dairy or dairy-beef) nested within study group. Calves’ centered birth weights, week of birth, age at vaccination (in days), and sex were considered fixed effects. The calves’ baseline virus antibody levels at Day −5 for each of the viruses being analyzed were included as covariates in ELISA OD unit models. Calf was included as a random effect and time since vaccination as a repeated measure. Tukey’s adjustment was included to account for unequal group sizes following study withdrawals. For all the analyses, significance was declared at a value of p of <0.05.

The frequency procedure (PROC FREQ) was used to report the non-normal distribution of categorical variables related to respiratory health scoring. To create binary data for analysis, health scoring categories were condensed into one overall cumulative pneumonia sign score. The respiratory health parameters that were scored and grouped into this aggregated pneumonia sign score were cough, respiratory score, demeanor, eyes and ocular discharge, ear position, nasal discharge, mobility, and interest in surroundings. Each calf was classified as having shown or not shown these aggregated pneumonia signs, meaning that a calf showed signs of severity of 1 or greater (from the four point scale; 0, 1, 2, 3) for two or more respiratory health parameters or showed signs of severity greater than 2 for an individual respiratory health parameter. To examine the effect of study group on rectal temperature, the data were also categorized into low (<38.5°C), normal (≥38.5°C – ≤ 39.5°C), and fever (>39.5°C).

PROC LOGISTIC was used to build a multinomial logistic regression model to determine the association between study groups, sex, breed classification, categorized rectal temperature, age in days at vaccination, and the aggregated pneumonia sign score. The controls were designated as the reference category for study group, female designated as the reference category for sex, and dairy as the reference category for breed class. The continuous variable rectal temperature was given a unit score of 0.5°C, and calf age at vaccination was also included in the model. The probability modeled was that showing two or more pneumonia signs >1 (binary, yes/no) and showing 1 pneumonia sign >2 was equal to 1. The likelihood of health events was reported as odds ratios (OD), with 95% confidence intervals (CIs).

## Results

### Environmental conditions

The average temperature in the calf houses throughout the period of housing was 9.3 ± 4.01°C; the average relative humidity was 81.3 ± 14.03%. The maximum air temperature in the calf houses was 23.6°C, the minimum air temperature was −1.0°C. The minimum and maximum relative humidity were 27 and 100%, respectively.

### Health scores

According to health scores, no adverse reactions were observed following concurrent vaccination (Table 1), the CV was not more likely to exhibit the statistically grouped pneumonia signs than CONT (OR = 0.975, CI = 0.534–1.779).

**Table 1:** Distribution frequencies (%) of individual and grouped health factor scores for each study group the days before and after IN vaccination*.

Parameter	Health score ≥ 1 per study group (%)							
	CONT		IO		RPO		CV	
	Period 1	Period 2	Period 1	Period 2	Period 1	Period 2	Period 1	Period 2
Cough	0	0	0	0	0	0	0	0
Nasal discharge	10.8	8.3	12.5	1.7	5.6	13.3	7.5	10
Ocular discharge	46.0	16.7	30.0	1.7	22.2	18.3	30.0	13.3
Ear position	8.1	15.0	10.0	16.7	5.6	15.0	7.5	10.0
Respiratory score	2.7	0	2.5	0	0	3.3	0	8.3
Demeanor	16.2	12.9	30.0	12.9	19.4	14.5	30.0	12.9
Mobility	2.7	13.3	5.0	10.0	11.1	16.7	5.0	11.7
Interest in surroundings	2.7	13.3	15.0	8.3	13.9	16.7	17.5	10.0
Aggregated pneumonia signs**	4.4	5.4	7.5	3.6	4.4	5.4	6.3	3.2

*Controls (CONT), vaccinated with just Bovilis IBR Marker Live® (IO), vaccinated with just Bovilis INtranasal RSP Live® (RPO), vaccinated concurrently with both Bovilis INtranasal RSP Live® and Bovilis IBR Marker Live® (CV), Period 1, clinically health scored for 3 days before intranasal (IN) vaccination; Period 2, clinically health scored for 6 days after IN vaccination. **Grouped pneumonia signs; meaning that calf showed signs of severity >1 (from the four point scale; 0, 1, 2, 3) for 2 or more of the respiratory health parameters; cough, nasal discharge, ocular discharge, ear position, respiratory score, demeanor, mobility, and interest in surroundings or else showed signs of severity >2 for an individual respiratory health parameter.

### Rectal temperatures

Study group did not have a significant effect on rectal temperatures (*p* = 0.676), although the vaccine delivery method was found to affect the likelihood of a fever occurring. While IN vaccination did not increase the likelihood that a calf would have a fever (OR = 1.189; CI = 0.700–1.994), post-parenteral vaccine delivery calves were 5.9 (CI = 3.610–9.752) times more likely to have a temperature > 39.5°C (*p* < 0.0001) than they were post-IN vaccination.

### Weights and average daily gains

The study group had a significant effect on average daily gains (ADGs) (*p* = 0.031; Table 2) from week 3 to week 18 post-IN vaccination, but there was no difference in ADGs (kg) between CV calves and CONT at any time point (Table 2).

**Table 2:** Adjusted average daily gain (kg) for each study group by time periods, with standard error mean (SEM) across all study groups.

Time period	Average daily gains (kg)					*p*-value (Study group)
	CONT	IO	RPO	CV	SEM	
On milk (aged 3–11 weeks)	0.70^a^	0.64^ab^	0.61^b^	0.67^ab^	0.026	0.077
Post-weaning (aged 12–15 weeks)	0.79^ab^	0.72^a^	0.75^ab^	0.85^b^	0.041	0.103
Post-parenteral vaccination (aged 16–18 weeks)	1.11	0.90	0.89	0.83	0.110	0.279
Start to finish (aged 3–18 weeks)	0.77^a^	0.69^b^	0.69^b^	0.74^ab^	0.023	0.031

On milk, weeks 1–7 on trial; post-weaning, weeks 8–12 on trial; post-parenteral vaccination, weeks 13–14 on trial; start to finish, weeks 1–14 on trial. Where study groups that share a letter differences are non-significant. Controls (CONT), vaccinated with just Bovilis IBR Marker Live® (IO), vaccinated with just Bovilis INtranasal RSP Live® (RPO), and vaccinated concurrently with both Bovilis INtranasal RSP Live® and Bovilis IBR Marker Live® (CV).

However, CONT gained more weight than IO and RPO calves, which were similar (Table 2). During the pre-weaning period, RPO gained on average 0.09 (±0.026) kg less per day than CONT (Table 2). Post-weaning IO gained 0.13 (± 0.041) kg less per day than CV (Table 2).

**Figure 1:**
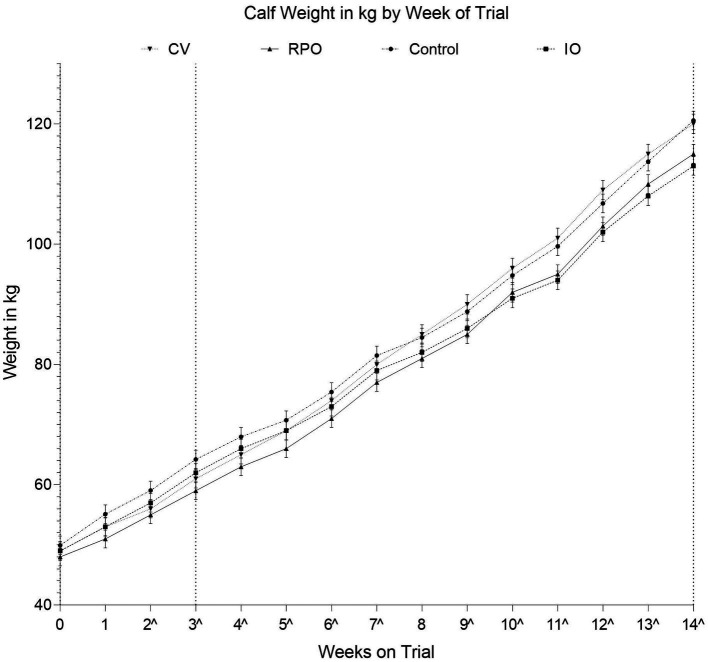
Adjusted average weights for each vaccination group by number of weeks on trial, calves were three weeks of age (22 ± 4.9 days of age) on the day of IN vaccination; Controls (CONT), vaccinated with just Bovilis IBR Marker Live (IO), vaccinated with just Bovilis INtranasal RSP Live (RPO), vaccinated concurrently with both Bovilis INtranasal RSP Live and Bovilis IBR Marker Live (CV). Dashed line on the *x*-axis indicates each post vaccination blood sampling event, ^ indicates weeks where groups were significantly different.

There was a significant study group by week interaction effect on calf weight (*p* = 0.026; Figure 1). However, the CV calves were of similar weight to the CONT calves across the duration of the study (*p* = 0.632; Figure 1).

### Virus-specific antibodies

**Figure 2:**
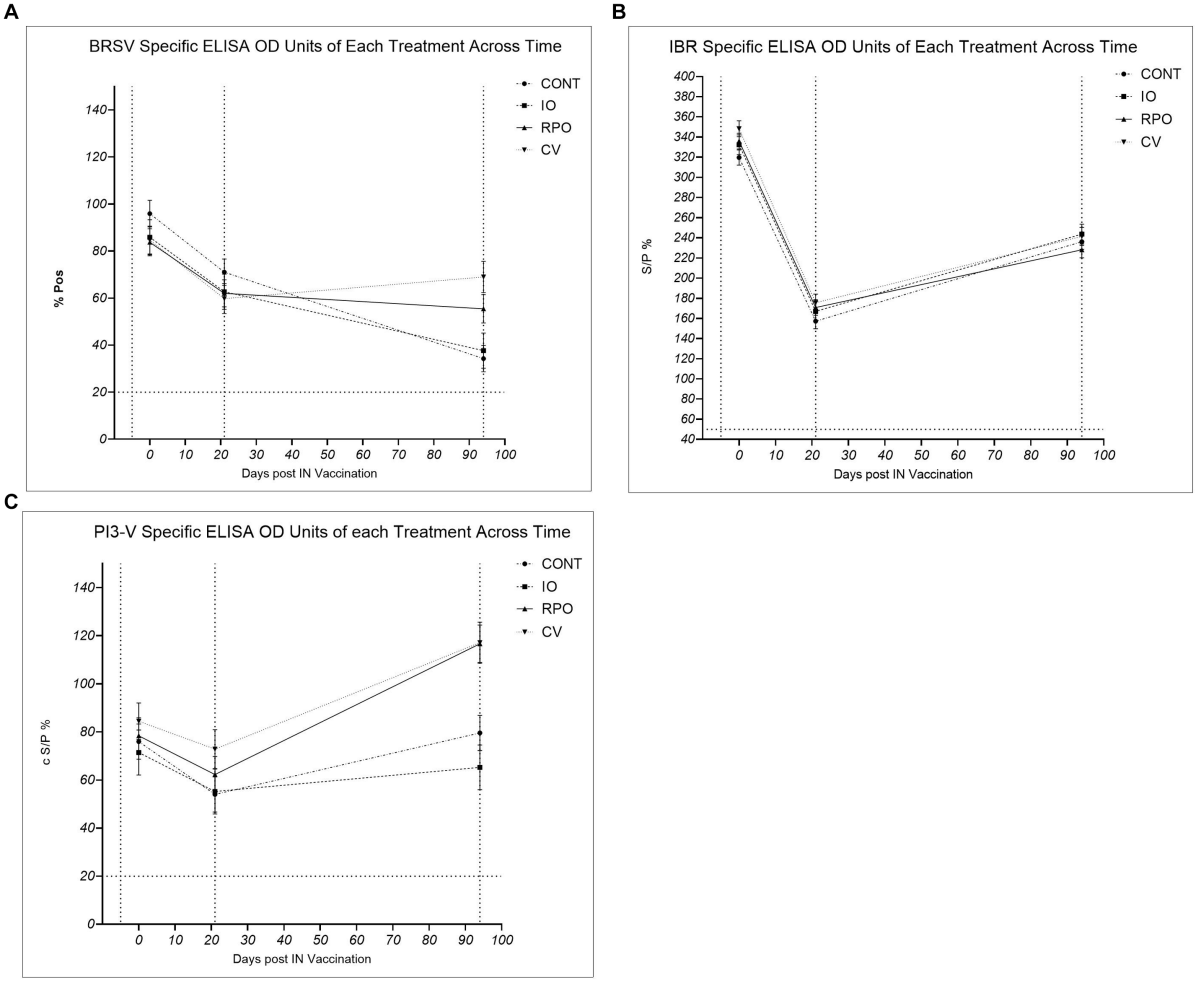
Bovine herpes virus type 1 (IBR), para-influenza type 3 (P13-V), and bovine respiratory syncytial virus (BRSV), Controls (CONT), vaccinated with just Bovilis IBR Marker Live® (IO), vaccinated with just Bovilis INtranasal RSP Live® (RPO), vaccinated concurrently with both Bovilis INtranasal RSP Live® and Bovilis IBR Marker Live® (CV). * c S/P % ≥ 20 = Positive, ** % *P* ≥ 10 = Positive, *** % S/P > 50 = Positive. Dashed line on the *x*-axis indicates each blood sampling event at Day -5, Day 21 and Day 94, dashed line on the y axis for seropositivity threshold. **(A)** BRSV %Pos *: Adjusted mean vaccination group ± SE corrected percent positive for BRSV antibodies at each blood sampling event. **(B)** IBR ** S/P %: Adjusted mean vaccination group ± SE antibody sample to positive ratio for IBR (BoHV-1) antibodies at each blooding event. **(C)** PI3-V c S/P %*: Adjusted mean vaccination group ± SE corrected sample to positive ratio for P13-V antibodies at each blood sampling event.

The study group had a significant effect on antibody levels against PI3-V (*p* = 0.003). While initially there were no significant differences across the study groups in antibody levels for PI3-V at Day 21 (*p* > 0.05), by Day 94, RPO and CV calves had significantly higher PI3-V antibodies than IO and CONT (*p* < 0.05; Figure 2). The study group was not significant for BRSV antibodies across the duration of the study (*p* = 0.640), but at Day 94, CV had significantly higher BRSV antibodies than the CONT (*p* = 0.0.011; Figure 2). Calves vaccinated for BRSV and PI3-V intranasally at 3 weeks of age (RPO and CV) showed a heightened response to both viruses following the parenteral BRSV/PI3-V vaccination when compared to CONT and IO, which were similar (Figure 2).

The study group did not have a significant effect on BoHV-1 antibody levels following IN or parenteral vaccination (Figure 2; *p* = 0.172). Due to the high levels of specific BoHV-1 antibodies on Day 0, subsequent IBR gE tests were also carried out on Day −5 and Day 0 sera. On Day 0, wild-type BoHV-1 virus antibodies were confirmed as present in calf sera; the IBR gE antibody levels also showed no significant difference between study groups for wild-type antibodies on Day −5 (*p* = 0.574) or Day 0 (*p* = 0.217).

## Discussion

### Serological response

We failed to accept the second hypothesis that concurrent vaccination would elicit a detectable serological response similar to individual vaccine administration at 21 days post-IN delivery, and the vaccines did not alter antibody levels in the calves at 3 weeks post-IN. It has been previously observed that vaccination in the face of maternally derived antibodies can fail to evoke a serological response but may induce an amnesic response upon later antigen exposure (36). There was no significant difference between the study groups for any of the three viruses at Day 21. Overall antibody levels fell for each of the viruses in all study groups, but this reduction in antibodies is in line with the observed rates of natural rates of maternally derived colostral antibody decay observed by Kirkpatrick et al. (42). It is likely that the presence of maternally derived antibodies masked any detectable emergence of serological responses to the IN vaccines; however, studies have shown that intranasal vaccines can be associated with the upregulation of genes leading to the induction of innate and specific cellular immune pathways (43). The protection conferred by maternal antibodies is somewhat counter-intuitive to the development of an adaptive immune response via vaccination, as the MDAs can bind the virus particles, blocking the development of B cell clonal expansion by the calves’ own immune system. Kirkpatrick et al. found that such maternally derived antibodies can persist for as long as 200 days for BRSV, > 65 days for BoHV-1, and > 183 days for PI3-V. Barry et al. also found that IBR, BRSV, and PI3-V derived from colostrum persisted for several months (41, 42).

The findings of the ELISA carried out are not entirely unique, and several other studies have observed that vaccinations of calves in the face of high levels of MDAs often fail to induce a serological response, but young calves immune systems can then show an amnesic effect upon re-encountering the virus particles (23, 44). In our study, this was seen as an apparent “primed” response to parenteral vaccination with BRSV and PI3-V in the calves that had received a prior IN vaccine (RPO and CV), which is because live intranasal vaccines likely first elicit a non-humoral predominantly cell-mediated response (23, 28). However, a humoral immune response was then seen on Day 94 when an improved or “primed” response to BRSV and PI3-V parenteral vaccines was seen in the CV and RPO study groups. Crucially, these antibody levels for BRSV and PI3-V were not significantly different between the RPO and CV.

The “primed” immune status seen on Day 94 in CV and RPO indicates that the intranasal vaccination set in motion an innate immune cascade response to the MLV at the site of infection. Upon recognition of a virus particle, the host immune system will produce a range of signaling chemokines and cytokines (45). This cascade response may not result in a detectable serological change; however, a prior study showed how it is sufficient to overcome a disease challenge (36). In this experiment and others, it induced improved responses to a parenteral vaccine dose (46). Mucosal immune signaling may not confer long-term immunity but potentially protects calves from the infection before they are old enough to develop a sustained immune response or to build an established immunological memory via a second vaccination. There are a number of studies which have been undertaken in other mammals that have shown how maternal antibody influence may impair the development of humoral responses but can act to improve the development of cell-mediated immunity and signaling (35, 47, 48). A potential explanation as to how the calves failed to show a serological response 3 weeks post-IN vaccination is due to the masking influence of maternally derived antibodies. This capacity of colostrum-derived antibodies to limit the efficacy of intranasal vaccination is well documented (36). Another study of similar methodology by Palomares et al. that involved intranasally vaccinating calves against BoHV-1, BRSV, and PI3-V, followed by the delivery of either IN or parenteral boosters, found similarly that an amnesic antibody response to IN vaccination will occur post parenteral booster delivery; interestingly, the study also found that subcutaneous booster vaccination induced significantly greater BRSV-specific antibody titers and IgA concentration compared to calves that received no prior IN vaccination (49). Based on these findings and the findings of current study, it would appear that the delivery of an IN vaccination to the neonate followed by a parenteral booster may be most effective vaccination strategy against BoHV-1, BRSV, and PI3-V.

An alternative strategy sometimes suggested to attempt to overcome the masking effect seen in calves with high MDA titers is to vaccinate the calf immediately post-birth before the colostral antibodies are absorbed and can take effect on the vaccine derived antigens. However, a 2023 study by Martínez et al. found that calves vaccinated intranasally at birth with an MLV BRSV vaccine showed similar responses to controls following a later disease challenge (50). Suggesting this practice is of no benefit. Intranasal vaccination several weeks post-colostrum followed by a parenteral booster post-weaning later may provide better protection, while a 2012 study by Hill et al. found that vaccinating calves intranasally at 0 and then again at 35 days of age with a MLV vaccine against bovine herpesvirus 1, bovine viral diarrhea virus 1, and bovine viral diarrhea virus 2 did induce IgA production (46). A study to further investigate the most optimal time of delivery of IN vaccination to the neonatal calf is perhaps warranted and should be a topic of further investigation.

The calves in this study had high initial levels of immunoglobulins to the viruses in question, which is likely because they received high quality colostrum, greater than 22% on the Brix refractometer, equivalent to 50 mg/mL (40, 51). Good colostrum management is a cornerstone of calf rearing, it must always prioritized to ensure optimum protection against infectious diseases in the young calf, regardless of a herd’s vaccination plan or disease status (39, 41).

Interestingly, IBR antibody levels remained similar across study groups in the weeks following IN vaccination and even at Day 94, i.e., post-parenteral vaccination administration. The IBR vaccination program of the herd from which the calves enrolled in the experiment were selected involved vaccinating the dams on average 4 weeks before calving, which may partly explain the high levels of antibodies observed in the calves at Day 0. Furthermore, our subsequent retesting of calf sera for BoHV-1 gE revealed that antibodies for wild-type IBR virus were also present in the calf sera at Day 0. Therefore, it is likely that the wild-type BoHV-1 antibodies present in the colostrum were of high enough levels to mask any response to the intranasal IBR vaccinations given to the IO and CV calves. In herds such as this with circulating infection, it is recommended that young stock are vaccinated prior to entering the mature herd. While intranasal vaccination does not always allow for a strong T-cell or humoral response, it does ensure targeted delivery of the antigen onto the mucosal surface, which is believed to confer protection directly at the site of pathogen entry (52).

Intranasal vaccination can also certainly play an additional role in priming calves for a second parenteral vaccination, helping to induce an improved endogenous humoral response as maternally derived antibodies decay. The ability of intranasal vaccines to also induce this mucosal immunity is one of their key advantages for use in young animals. Woolums et al. found that IN vaccination against BRSV with MLV resulted in increased IFN-γ levels (a key immune signaling cytokine) following an induced BRSV challenge (37). While this trial contained no challenge component to verify vaccine efficacy, another IN vaccine study with similar methodology showed that IN MLV vaccinations can trigger localized mucosal innate immune responses (31). We would postulate that additional testing for inflammatory modulators, e.g., IFN-α and IFN-γ, may have revealed a detectable innate immune response to the IN vaccinations (37, 53, 54).

### Effect of vaccination on average daily weight gain

We failed to reject the hypothesis that CV vaccination would have no effect on weight, while initially it might have been expected that vaccination would result in improved weight gain by reducing infection associated with ill thrift. Average daily gains were the same in CV and CONT. Therefore, both CV and CONT mean ADGs did meet the recommended target of 0.70 kg/day for pre-weaning average daily weight gain in dairy and dairy-beef calves (55, 56). The IO and RPO calves gained significantly less weight than the CONT. This is an interesting finding as it would suggest that the concurrent vaccination had no effect on weight gain but that vaccination with the vaccine for BoHV-1 or BRSV/PI3-V alone may have resulted in poorer growth performances. The reason for this difference remains unclear and perhaps warrants further investigation. Several other studies have also found that pneumonia virus vaccination of calves may not result in improved weight gains in calves (57–59). It is possible that episodes of mild scours that, across all treatment groups during the trial, were also in part responsible for these generally poor growth rates. In addition, as three calves were withdrawn from the study (two animals from CONT group and one from the RPO group), before completion due to unrelated gastrointestinal illnesses, it is also possible that the experiment was under powered, meaning any potential differences in weight gain not seen were due to the reduced group sizes. While this study has shown that the initial challenge of the modified live vaccine viruses may have impaired calf weight gain in the short term, it is perhaps worth balancing this against the risks to animal welfare and performance that can be posed through the onset of an acute infection, if animals are to remain naïve and contract the virus(es).

It is well documented that respiratory disease outbreaks have long-term implications on animal performance. It has also been suggested that subclinical pneumonia is often an underlying cause for ill thrift in both dairy and beef animals (60). Failing to reach target weights has long-term implications on heifer fertility and production performance (13, 61). Poor health of course reduces calf weight gain both directly through the metabolic burden of fighting an infection, be it acute or chronic, and indirectly as symptomatic onset will reduce calf concentrate and milk intake. Post-mortem studies may also find many animals that have experienced pneumonia in calfhood will present with lesions at slaughter, suggestive of a lifetime of chronic infection. Vaccination of the young animals could offer a protective effect against the development of this state of impaired growth due to persistent infection.

### Pneumonia signs

Concurrently vaccinated calves were found to be no more likely to exhibit signs of pneumonia in the days after vaccination than CONT. The rectal temperature results also showed that the calves did not experience a fever response to the intranasal vaccines. While this study involved a small sample size of 10 calves, on a single farm, no adverse effects were noticed following vaccination. The lack of significant differences in health scores, pneumonia signs, and rectal temperatures would suggest that it is likely safe to administer Bovilis IBR Marker Live only® and Bovilis Intranasal RSP Live® simultaneously.

### Future implications

The intranasal concurrent vaccination resulted in a successful “priming” response to subsequent BRSV and PI3-V antigen exposure. This “priming” was demonstrated by the anamnestic antigen responses seen following the administration of a subsequent parenteral vaccine. This is an important finding as it relates directly to calves vaccinated in the face of MDAs. If trials for licensing vaccine efficacy are carried out on colostrum-deprived seronegative calves, they are not directly applicable to on-farm practices as calves receiving colostrum will be subject to this interference by MDAs. However, the findings of this study would suggest that IN vaccination may be effective in priming calves for an improved serological response to a parental injection and that the delivery of both vaccines simultaneously is likely safe. The finding also suggests that both intranasal vaccines could safely be delivered simultaneously from as young as 3 weeks of age, an important consideration for ease of animal handling and, additionally, if a farmer/veterinarian is opting to vaccinate in the face of an outbreak if the causative viral agent(s) of an infectious outbreak are unknown.

The aim of a vaccination program is not only to reduce the spread of disease but also to critically limit viral shedding in already infected animals (62, 63). It is recommended, for example, that dairy replacement heifer calves should undergo a vaccination plan in their first year of life to avoid entering the herd naïve to the viruses already circulating in the mature cow herd. If heifers contract these viruses either as young calves or when entering the milking herd, it can lead to reduced milk yields. Intranasal vaccination may be crucial in reducing dependency on antimicrobials in the treatment of respiratory infections, with pneumonia still being one of the main reasons for their use in pre-weaned calves (18, 62). Given that recent studies have isolated multidrug-resistant *Mannheimia haemolytica* from high-risk beef stocker cattle after antimicrobial metaphylaxis, it is important that farmers and vets employ strategies to limit this dependency on antimicrobials (64). Vaccination when combined with optimized colostrum management, nutrition, and housing should act to reduce the incidence of respiratory infections in pre-weaned calves.

## Conclusion

The results of this field trial suggest that calves vaccinated intranasally via concurrent administration of IBR Marker Live® and Bovilis INtranasal RSP Live® at 3 weeks of age can develop an amnestic response to BRSV and PI3-V antigens. This “priming” response was observed following a second exposure to the virus antigens in a parenteral vaccination at 15 weeks of age, which indicates that intranasal followed by parental vaccination improved immunity to BRSV and PI3-V in pre-weaned dairy and dairy-beef calves as compared to the CONT calves that were given only a delayed parental vaccination. Additionally, it could result in improved effectiveness of parental pneumonia vaccines given subsequently.

## Data availability statement

The raw data supporting the conclusions of this article will be made available by the authors, upon request.

## Ethics statement

The animal study was approved by ethical approval to complete the study was granted by the Teagasc Animal Ethics Committee (TAEC2020-278), and a license was granted by the Health Products Regulatory Authority (Clinical Field Trial License Number: CT10452/002). The study was conducted in accordance with the local legislation and institutional requirements.

## Author contributions

AF: Formal analysis, Investigation, Methodology, Writing – original draft, Data curation, Project administration. CaM: Conceptualization, Methodology, Supervision, Writing – review & editing. KS: Methodology, Project administration, Resources, Writing – review & editing. RF: Project administration, Resources, Writing – review & editing. CS: Methodology, Writing – review & editing. BC: Methodology, Funding acquisition, Writing – review & editing. CoM: Supervision, Writing – review & editing. EK: Conceptualization, Data curation, Formal analysis, Funding acquisition, Investigation, Methodology, Project administration, Resources, Supervision Visualization, Writing – review & editing.

## References

[1] Dubrovsky SA van Eenennaam AL Karle BM Rossitto PV Lehenbauer TW Aly SS. Bovine respiratory disease (BRD) cause-specific and overall mortality in preweaned calves on California dairies: the BRD 10K study. J Dairy Sci. (2019) 102:7320–8. doi: 10.3168/jds.2018-15463, PMID: 31202642

[2] Taylor JD Fulton RW Lehenbauer TW Step DL Confer AW. The epidemiology of bovine respiratory disease: what is the evidence for predisposing factors? Can Vet J. (2010) 51:1351–9. 21197200 PMC2942046

[3] Panciera RJ Confer AW. Pathogenesis and pathology of bovine pneumonia. Vet Clin. (2010) 26:191–214. doi: 10.1016/j.cvfa.2010.04.001, PMID: 20619179 PMC7185769

[4] Department of Agriculture, Food and the Marine of Ireland, Agri-Food & Biosciences Institute, Northern Ireland. (2021). Available at: http://www.animalhealthsurveillance.agriculture.gov.ie/

[5] Ollivett TL Buczinski S. On-farm use of ultrasonography for bovine respiratory disease. Vet Clin. (2016) 32:19–35. doi: 10.1016/j.cvfa.2015.09.001, PMID: 26922110

[6] Ellis JA. Update on viral pathogenesis in BRD. Anim Health Res Rev. (2009) 10:149–53. doi: 10.1017/S146625230999020X, PMID: 20003652

[7] Gaudino M Nagamine B Ducatez MF Meyer G. Understanding the mechanisms of viral and bacterial coinfections in bovine respiratory disease: a comprehensive literature review of experimental evidence. Vet Res. (2022) 53:70. doi: 10.1186/s13567-022-01086-1, PMID: 36068558 PMC9449274

[8] Blakebrough-Hall C McMeniman JP González LA. An evaluation of the economic effects of bovine respiratory disease on animal performance, carcass traits, and economic outcomes in feedlot cattle defined using four BRD diagnosis methods. J Anim Sci. (2020) 98:skaa005. doi: 10.1093/jas/skaa005 31930299 PMC6996507

[9] Cuevas-Gómez I McGee M Sánchez JM O’Riordan E Byrne N McDaneld T. Association between clinical respiratory signs, lung lesions detected by thoracic ultrasonography and growth performance in pre-weaned dairy calves. Ir Vet J. (2021) 74:1–9. doi: 10.1186/s13620-021-00187-1 33766106 PMC7992334

[10] McGill JL Sacco RE. The immunology of bovine respiratory disease: recent advancements. Vet Clin North Am Food Anim Pract. (2020) 36:333–48. doi: 10.1016/j.cvfa.2020.03.002, PMID: 32327252 PMC7170797

[11] van der Fels-Klerx HJ Saatkamp HW Verhoeff J Dijkhuizen AA. Effects of bovine respiratory disease on the productivity of dairy heifers quantified by experts. Livest Prod Sci. (2002) 75:157–66. doi: 10.1016/S0301-6226(01)00311-6

[12] Bach A. Associations between several aspects of heifer development and dairy cow survivability to second lactation. J Dairy Sci. (2011) 94:1052–7. doi: 10.3168/jds.2010-3633 21257075

[13] Dunn TR Ollivett TL Renaud DL Leslie KE LeBlanc SJ Duffield TF. The effect of lung consolidation, as determined by ultrasonography, on first-lactation milk production in Holstein dairy calves. J Dairy Sci. (2018) 101:5404–10. doi: 10.3168/jds.2017-13870 29525311

[14] Delabouglise A James A Valarcher JF Hagglünd S Raboisson D Rushton J. Linking disease epidemiology and livestock productivity: the case of bovine respiratory disease in France. PLoS One. (2017) 12:e0189090. doi: 10.1371/journal.pone.0189090, PMID: 29206855 PMC5716546

[15] Fernández M Ferreras MC Giráldez FJ Benavides J Pérez V. Production significance of bovine respiratory disease lesions in slaughtered beef cattle. Animals. (2020) 10:1770. doi: 10.3390/ani10101770, PMID: 33007901 PMC7599887

[16] Wilson BK Step DL Maxwell CL Gifford CA Richards CJ Krehbiel CR. Effect of bovine respiratory disease during the receiving period on steer finishing performance, efficiency, carcass characteristics, and lung scores. Prof Anim Sci. (2017) 33:24–36. doi: 10.15232/pas.2016-01554, PMID: 32288478 PMC7147665

[17] Bokma J Boone R Deprez P Pardon B. Risk factors for antimicrobial use in veal calves and the association with mortality. J Dairy Sci. (2019) 102:607–18. doi: 10.3168/jds.2018-15211, PMID: 30415845

[18] Kuipers A Koops WJ Wemmenhove H. Antibiotic use in dairy herds in the Netherlands from 2005 to 2012. J Dairy Sci. (2016) 99:1632–48. doi: 10.3168/jds.2014-8428, PMID: 26709178

[19] Pardon B Catry B Dewulf J Persoons D Hostens M de Bleecker K. Prospective study on quantitative and qualitative antimicrobial and anti-inflammatory drug use in white veal calves. J Antimicrob Chemother. (2012) 67:1027–38. doi: 10.1093/jac/dkr570, PMID: 22262796

[20] Sawant AA Sordillo LM Jayarao BM. A survey on antibiotic usage in dairy herds in Pennsylvania. J Dairy Sci. (2005) 88:2991–9. doi: 10.3168/jds.S0022-0302(05)72979-9, PMID: 16027213

[21] Mathew AG Cissell R Liamthong S. Antibiotic resistance in bacteria associated with food animals: a United States perspective of livestock production. Foodborne Pathog Dis. (2007) 4:115–33. doi: 10.1089/fpd.2006.0066, PMID: 17600481

[22] van Boeckel TP Brower C Gilbert M Grenfell BT Levin SA Robinson TP. Global trends in antimicrobial use in food animals. Proc Natl Acad Sci. (2015) 112:5649–54. doi: 10.1073/pnas.1503141112, PMID: 25792457 PMC4426470

[23] Chamorro MF Woolums A Walz PH. Vaccination of calves against common respiratory viruses in the face of maternally derived antibodies (IFOMA). Anim Health Res Rev. (2016) 17:79–84. doi: 10.1017/S1466252316000013, PMID: 27039687

[24] Fulton RW. Bovine respiratory disease research (1983–2009). Anim Health Res Rev. (2009) 10:131–9. doi: 10.1017/S146625230999017X, PMID: 20003649

[25] Urban-Chmiel R Grooms D. Prevention and control of bovine respiratory disease. J Livestock Sci. (2012) 3:27–36.

[26] Woolums A.R. Vaccinating calves. In American Association of Bovine Practitioners Proceedings of the annual conference. Vancouver, British Columbia: Frontier Printers, Inc. (2007).

[27] Smith DR. Risk factors for bovine respiratory disease in beef cattle. Anim Health Res Rev. (2020) 21:149–52. doi: 10.1017/S1466252320000110, PMID: 33682661

[28] Chase CC Hurley DJ Reber AJ. Neonatal immune development in the calf and its impact on vaccine response. Vet Clin N Am Food Anim Pract. (2008) 24:87–104. doi: 10.1016/j.cvfa.2007.11.001, PMID: 18299033 PMC7127081

[29] Chase CCL. Acceptable young calf vaccination strategies—what, when, and how? The veterinary clinics of North America. Food Anim Pract. (2022) 38:17–37. doi: 10.1016/j.cvfa.2021.11.002, PMID: 35219483

[30] Barrington GM Parish SM. Bovine neonatal immunology. Vet Clin N Am Food Anim Pract. (2001) 17:463–76. doi: 10.1016/s0749-0720(15)30001-3 PMID: 11692503 PMC7135619

[31] Chamorro MF Palomares RA. Bovine respiratory disease vaccination against viral pathogens: modified-live versus inactivated antigen vaccines, intranasal versus parenteral, what is the evidence? Vet Clin Food Anim Pract. (2020) 36:461–72. doi: 10.1016/j.cvfa.2020.03.006, PMID: 32451035 PMC7244452

[32] Cortese VS. Neonatal immunology. Vet Clin N Am Food Anim Pract. (2009) 25:221–7. doi: 10.1016/j.cvfa.2008.10.003 PMC7126137 19174291

[33] Bryson G Adair BM McNulty MS McAliskey M Bradford HEL Allan GM. Studies on the efficacy of intranasal vaccination for the prevention of experimentally induced parainfluenza type 3 virus pneumonia in calves. Vet Rec. (1999) 145:33–9. doi: 10.1136/vr.145.2.33, PMID: 10458574

[34] Vangeel I Antonis AFG Fluess M Riegler L Peters AR Harmeyer SS. Efficacy of a modified live intranasal bovine respiratory syncytial virus vaccine in 3-week-old calves experimentally challenged with BRSV. Vet J. (2007) 174:627–35. doi: 10.1016/j.tvjl.2006.10.013, PMID: 17169592

[35] Meganck V Opsomer G Piepers S Cox E Goddeeris BM. Maternal colostral leukocytes appear to enhance cell-mediated recall response, but inhibit humoral recall response in prime–boost vaccinated calves. J Reprod Immunol. (2016) 113:68–75. doi: 10.1016/j.jri.2015.11.004, PMID: 26796988

[36] Windeyer MC Gamsjäger L. Vaccinating calves in the face of maternal antibodies: challenges and opportunities. Vet Clin Food Anim Pract. (2019) 35:557–73. doi: 10.1016/j.cvfa.2019.07.004, PMID: 31590902

[37] Woolums AR Brown CC Brown JC Jr Cole DJ Scott MA Williams SM. Effects of a single intranasal dose of modified-live bovine respiratory syncytial virus vaccine on resistance to subsequent viral challenge in calves. Am J Vet Res. (2004) 65:363–72. doi: 10.2460/ajvr.2004.65.363, PMID: 15027687

[38] Barry J Kennedy E Sayers R De Boer IJM Bokkers EAM. Development of a welfare assessment protocol for dairy calves from birth through to weaning. Colostrum Feeding Calf Welfare Assessment. (2019) 28:331–344. doi: 10.7120/09627286.28.3.331

[39] Barry J Bokkers EAM Berry DP de Boer IJM McClure J Kennedy E. Associations between colostrum management, passive immunity, calf-related hygiene practices, and rates of mortality in preweaning dairy calves. J Dairy Sci. (2019) 102:10266–76. doi: 10.3168/jds.2019-16815, PMID: 31521357

[40] Bielmann V Gillan J Perkins NR Skidmore AL Godden S Leslie KE. An evaluation of brix refractometry instruments for measurement of colostrum quality in dairy cattle. J Dairy Sci. (2010) 93:3713–21. doi: 10.3168/jds.2009-2943, PMID: 20655440

[41] Barry J Bokkers EAM Sayers R Murphy JP de Boer IJM Kennedy E. Effect of feeding single-dam or pooled colostrum on maternally derived immunity in dairy calves. J Dairy Sci. (2022) 105:560–71. doi: 10.3168/jds.2021-20343, PMID: 34763911

[42] Kirkpatrick J Fulton RW Burge LJ DuBois WR Payton M. Passively transferred immunity in newborn calves, rate of antibody decay, and effect on subsequent vaccination with modified live virus vaccine. Bovine Pract. (2019) 35:47–55. doi: 10.21423/bovine-vol35no1p47-55

[43] Nuijten P Cleton N van der Loop J Makoschey B Pulskens W Vertenten G. Early activation of the innate immunity and specific cellular immune pathways after vaccination with a live intranasal viral vaccine and challenge with bovine parainfluenza type 3 virus. Vaccine. (2022) 10:104. doi: 10.3390/vaccines10010104, PMID: 35062765 PMC8777984

[44] Menanteau-Horta A Ames TR Johnson DW Meiske JC. Effect of maternal antibody upon vaccination with infectious bovine rhinotracheitis and bovine virus diarrhea vaccines. Can J Comp Med. (1985) 49:10–4. PMID: 2985214 PMC1236109

[45] Firth MA Shewen PE Hodgins DC. Passive and active components of neonatal innate immune defenses. Anim Health Res Rev. (2005) 6:143–58. doi: 10.1079/AHR2005107, PMID: 16583779

[46] Hill KL Hunsaker BD Townsend HG van Drunen Littel-van den Hurk S Griebel PJ. Mucosal immune response in newborn Holstein calves that had maternally derived antibodies and were vaccinated with an intranasal multivalent modified-live virus vaccine. J Am Vet Med Assoc. (2012) 240:1231–40. doi: 10.2460/javma.240.10.1231 22559114

[47] Williams PP. Immunomodulating effects of intestinal absorbed maternal colostral leukocytes by neonatal pigs. Can J Vet Res. (1993) 57:1–8. PMID: 8431798 PMC1263580

[48] Tuboly S Bernáth S Glávits R Kovács A Megyeri Z. Intestinal absorption of colostral lymphocytes in newborn lambs and their role in the development of immune status. Acta Vet Hung. (1995) 43:105–15. PMID: 7625282

[49] Palomares RA Bittar JHJ Woolums AR Hoyos-Jaramillo A Hurley DJ Saliki JT. Comparison of the immune response following subcutaneous versus intranasal modified-live virus booster vaccination against bovine respiratory disease in pre-weaning beef calves that had received primary vaccination by the intranasal route. Vet Immunol Immunopathol. (2021) 237:110254. doi: 10.1016/j.vetimm.2021.110254, PMID: 34034143

[50] Martínez DA Chamorro MF Passler T Huber L Walz PH Thoresen M. Local and systemic antibody responses in beef calves vaccinated with a modified-live virus bovine respiratory syncytial virus (BRSV) vaccine at birth following BRSV infection. Vet Sci. (2023) 10:20. doi: 10.3390/vetsci10010020 PMC9863489 36669022

[51] Buczinski S Fecteau G Chigerwe M Vandeweerd JM. Diagnostic accuracy of refractometer and brix refractometer to assess failure of passive transfer in calves: protocol for a systematic review and meta-analysis. Anim Health Res Rev. (2016) 17:3–8. doi: 10.1017/S1466252316000074 27427188

[52] Correa VA Portilho AI De Gaspari E. Vaccines, adjuvants and key factors for mucosal immune response. Immunology. (2022) 167:124–38. doi: 10.1111/imm.13526, PMID: 35751397

[53] Kimman T Westenbrink F Straver P. Priming for local and systemic antibody memory responses to bovine respiratory syncytial virus: effect of amount of virus, virus replication, route of administration and maternal antibodies. Vet Immunol Immunopathol. (1989) 22:145–60. doi: 10.1016/0165-2427(89)90057-3, PMID: 2530685

[54] Midla LT Hill KL van Engen NK Edmonds M Renter DG Streeter MN. Innate and acquired immune responses of colostrum-fed neonatal Holstein calves following intranasal vaccination with two commercially available modified-live virus vaccines. J Am Vet Med Assoc. (2021) 258:1119–29. doi: 10.2460/javma.258.10.1119, PMID: 33944597

[55] Teagasc. Teagasc Beef Manual. (2016). Teagasc Agriculture and Food Development Authority.

[56] Teagasc. Teagasc Calf Rearing Manual. Teagasc Agriculture and Food Development Authority. (2017).

[57] Cho YS Lee HS Lim SK Joo YS Kim JM Kim JH. Safety and efficacy testing of a novel multivalent bovine bacterial respiratory vaccine composed of five bacterins and two immunogens. J Vet Med Sci. (2008) 70:959–64. doi: 10.1292/jvms.70.959, PMID: 18840971

[58] Lorenz I Earley B Gilmore J Hogan I Kennedy E More SJ. Calf health from birth to weaning. III. Housing and management of calf pneumonia. Ir Vet J. (2011) 64:14. doi: 10.1186/2046-0481-64-14, PMID: 22018053 PMC3220626

[59] Windeyer MC Leslie KE Godden SM Hodgins DC Lissemore KD LeBlanc SJ. The effects of viral vaccination of dairy heifer calves on the incidence of respiratory disease, mortality, and growth. J Dairy Sci. (2012) 95:6731–9. doi: 10.3168/jds.2012-5828, PMID: 22959931

[60] Griffin D. The monster we don't see: subclinical BRD in beef cattle. Anim Health Res Rev. (2014) 15:138–41. doi: 10.1017/S1466252314000255, PMID: 25497500

[61] Archbold H Shalloo L Kennedy E Pierce KM Buckley F. Influence of age, body weight and body condition score before mating start date on the pubertal rate of maiden Holstein–Friesian heifers and implications for subsequent cow performance and profitability. Animal. (2012) 6:1143–51. doi: 10.1017/S1751731111002692, PMID: 23031476

[62] Potter A Gerdts V den Hurk SD L-v. Veterinary vaccines: alternatives to antibiotics? Anim Health Res Rev. (2008) 9:187–99. doi: 10.1017/S1466252308001606 19102790

[63] Sherwin G Down P. Calf immunology and the role of vaccinations in dairy calves. In Pract. (2018) 40:102–14. doi: 10.1136/inp.k952

[64] Woolums AR Karisch BB Frye JG Epperson W Smith DR John Blanton FA Jr. Multidrug resistant Mannheimia haemolytica isolated from high-risk beef stocker cattle after antimicrobial metaphylaxis and treatment for bovine respiratory disease. Vet Microbiol. (2018) 221:143–52. doi: 10.1016/j.vetmic.2018.06.005 29981701

